# Identification of the Primary Structure of Selenium-Containing Polysaccharides Selectively Inhibiting T-Cell Proliferation

**DOI:** 10.3390/molecules26175404

**Published:** 2021-09-06

**Authors:** Marzenna Klimaszewska, Sabina Górska, Grzegorz Łapienis, Beata Kaleta, Sandra Górska, Marta Kaszowska, Maciej Dawidowski, Andrzej Gamian, Radoslaw Zagożdżon, Andrzej Górski, Jadwiga Turło

**Affiliations:** 1 Department of Drug Technology and Pharmaceutical Biotechnology, Medical University of Warsaw, Banacha 1, 02-097 Warsaw, Poland; marzenna.klimaszewska@wum.edu.pl (M.K.); sandra.gorska@wum.edu.pl (S.G.); maciej.dawidowski@wum.edu.pl (M.D.); 2 Microbiome Immunobiology Laboratory, Hirszfeld Institute of Immunology and Experimental Therapy, Polish Academy of Sciences, Weigla 12, 53-114 Wroclaw, Poland; sabina.gorska@hirszfeld.pl; 3 Division of Polymers, Centre of Molecular and Macromolecular Studies, Polish Academy of Sciences, Sienkiewicza 112, 90-363 Łodz, Poland; lapienis@cbmm.lodz.pl; 4 Department of Clinical Immunology, Medical University of Warsaw, Nowogrodzka 59, 02-006 Warsaw, Poland; beata.kaleta@wum.edu.pl (B.K.); radoslaw.zagozdzon@wum.edu.pl (R.Z.); 5 Laboratory of Microbial Immunochemistry and Vaccines, Hirszfeld Institute of Immunology and Experimental Therapy, Polish Academy of Sciences, Weigla 12, 53-114 Wroclaw, Poland; marta.kaszowska@hirszfeld.pl; 6 Medical Microbiology Laboratory, Hirszfeld Institute of Immunology and Experimental Therapy, Polish Academy of Sciences, Weigla 12, 53-114 Wroclaw, Poland; andrzej.gamian@hirszfeld.pl; 7 Bacteriophage Laboratory, Hirszfeld Institute of Immunology and Experimental Therapy, Polish Academy of Sciences, Weigla 12, 53-114 Wroclaw, Poland; andrzej.gorski@hirszfeld.pl

**Keywords:** *Lentinula edodes*, Se-containing polysaccharide, immunosuppressant, T lymphocyte

## Abstract

We previously described the biosynthesis, isolation, and immunosuppressive activity of the selenium-containing polysaccharide fraction isolated from the mycelial culture of *Lentinula edodes*. Structural studies have shown that the fraction was a protein-containing mixture of high molar mass polysaccharides α- and β-glucans. However, which of the components of the complex fraction is responsible for the immunosuppressive activity non-typical for polysaccharides of fungal origin has not been explained. In the current study, we defined four-polysaccharide components of the Se-containing polysaccharide fraction determined their primary structure and examined the effect on T- and B-cell proliferation. The isolated Se-polysaccharides, α-1,4-glucan (M_w_ 2.25 × 10^6^ g/mol), unbranched β-1,6-d-glucan, unbranched β-1,3-d-glucan and β-1,3-branched β-1,6-d-glucan (M_w_ 1.10 × 10^5^ g/mol), are not typical as components of the cell wall of *L. edodes*. All are biologically active, but the inhibitory effect of the isolated polysaccharides on lymphocyte proliferation was weaker, though more selective than that of the crude fraction.

## 1. Introduction

Mushroom-derived polysaccharides (MPSs), particularly β-d-glucans, are of great interest as immune-modulating substances recognized by the human immune system [1,2,3]. Most biologically active MPSs are extracted from the mushroom cell wall, which is composed of inner layers of chitin and β-d-glucans [4,5]. The cell wall β-d-glucans are typically 1,3- and 1,6-linked chains with varying amounts of side chains in position *O*-6 or *O*-3 [6,7,8].

Such a structure is described for lentinan, a highly purified polysaccharide fraction extracted from *Lentinula edodes* (shiitake mushroom) fruiting bodies. This substance is a highly potent enhancer of the immune system [9,10] approved for use in cancer treatment as an adjunct to conventional therapy [11,12,13]. However, the β-1,6; β-1,3-structure of cell wall MPSs appears to not be universal among Basidiomycota, as *Albatrellus ovinus* does not contain this type of β-d-glucan [14]. Furthermore, strain variation, developmental stage, culture method and conditions, medium composition, extraction method, and even drying method may result in variability in the monosaccharide composition and combinations among polysaccharides [15,16,17,18,19].

The most frequently mentioned parameters of the structure of polysaccharides showing immunomodulatory effect include molecular weight, monosaccharide composition, type of glycosidic bond, branching degree, conformation of the molecule and modification in the structure of the molecule (e.g., by esterification, phosphorylation, incorporation of element into the molecule) [20,21,22,23,24,25,26]. Detailed structural characterisation of immunoactive polysaccharides is challenging; however, probably the highest immunomodulatory activity shows the polymers of glucose and mannose, with a predominance of β-(1-3) bonds in the main chain, without too low or too high degrees of branching of the molecule (0.2–0.33) and with high molecular weight [27].

One of the methods used to increase the immunomodulatory activity of the polysaccharides by modifying their structure, is the incorporation of selenium in the molecules. The great interest in selenium-containing polysaccharides results from the expected synergy between selenium and polysaccharides [26,27,28]. However, the natural Se-polysaccharides are not common and the selenium content in them is very low [26]. Therefore, there is a growing interest in the chemical or biotechnological modification of polysaccharides to raise the concentration of selenium in them. In biotechnological methods, the ability of organisms to absorb the microelements present in the culture medium has been used; the culture medium is enriched with inorganic selenium (e.g., sodium selenite), leading to the incorporation of the element into the biopolymer structure and turning it into an organic form [26,27].

In our previous research, we obtained by biotechnological method a selenium-containing polysaccharide that was an analog of lentinan [27]. For this purpose, we used *L. edodes* mycelial cultures, grown under submerged conditions in a liquid medium enriched with sodium selenite [27,28,29]. We then extracted from the selenium-enriched mycelium the selenopolysaccharide fraction corresponding to lentinan by using one of the methods employed for the isolation of lentinan [27]. We expected that the incorporation of selenium into the polysaccharide would enhance the immunostimulatory activity of the isolated fraction compared to lentinan. However, the results of the preliminary biological tests of the obtained fraction were unexpected. The isolated Se-polysaccharide was a selective immunosuppressant with strong antioxidant activity and enhanced cell viability that was non-toxic [30]. The biological effect was completely different from lentinan. Preliminary structural studies have shown that the isolated immuno-active fraction is a protein-containing mixture of high molar mass polysaccharides (M_w_ 3.9 × 10^6^ g/mol and 2.6 × 10^5^ g/mol) α- and β-glucans are composed of glucose or mannose. 

The X-ray absorption fine structure (XAFS) spectral analysis in the near edge region (XANES) confirmed that in the Se-polysaccharides, selenium is present at the II oxidation state and organically bound. Simulation analysis in the XAFS region suggested that selenium is most likely bound by a glycosidic-link in a β-1,3 or α-1,4-glycosidic bond, or substituted for oxygen in a pyranosic ring [27]. However, which of the components of the complex fraction was responsible for the immunosuppressive activity—non-typical for polysaccharides of fungal origin—has not been determined. We hypothesized that the biological effect depends, in part, on the selenium incorporation into the polysaccharide molecule but likely also on the dissimilar structure of the fractions isolated from mycelial cultures versus lentinan. Thus, the current study aimed to separate pure polysaccharides from the Se-containing polysaccharide fraction isolated from the *L. edodes* mycelium, determine their primary structures, and investigate if and which of them was responsible for inhibiting T-cell proliferation.

## 2. Results

### 2.1. Composition of the Se-Le-30 (Se-Containing Lentinan Analog Isolated from Se-Enriched L. edodes Mycelium by the Chihara Method) Polysaccharide Fraction

#### 2.1.1. Selenium Content

The selenium concentration in Se-enriched mycelium cultivated in medium enriched with 30 µg Se/mL was 1753 μg/g. The concentration of selenium in the *Se-Le-30* isolated using the Chihara method was 48 μg/g. The concentration of selenium in the polysaccharide fractions purified by chromatographic methods is described in Section 2.2. was: fraction I—17 μg/g, fraction II—22 μg/g, and fraction III—38 μg/g.

#### 2.1.2. Monosaccharide Composition

The monosaccharide composition of the Se-enriched fraction, *Se-Le-30,* was determined after complete hydrolysis using trifluoroacetic acid. As with the previously described *Se-L (*Se-containing lentinan analog isolated from Se-enriched *L. edodes* mycelium by the Ng and Yap method) fraction [27], mannose and glucose constituted 99.5% of total monosaccharides by weight. However, the mannose content in the *Se-Le-30* fraction was lower than for the previously described *Se*-*L* fraction (6% versus 14%).

#### 2.1.3. Protein Content

The total protein content in the *Se-Le-30* fraction was 3.3% when determined by the Bradford method, nearly twofold lower than in the *Se*-*L* fraction isolated by Ng and Yap method (8.4%) [27]. The protein content in the *Se-Le-30* fraction, calculated based on the elemental analysis and conversion of total nitrogen to true protein, was similar to the Bradford method (4.8% for *Se-Le-30* and 10.2% for *Se-L)*. The use of conversion factors for total nitrogen to true protein is controversial, and considerable variation has been found in the conversion factors for different foods [31]. We assumed that the isolated polysaccharide fraction was free of impurities containing nitrogen and applied a conversion factor of 6.25.

### 2.2. Structural Analysis on Separated Polysaccharides of Se-Le-30 Crude Extract

The Se-containing polysaccharide crude extract (*Se-Le-30*) was purified by ion-exchange chromatography on diethylaminoethyl (DEAE)-Sephadex A-25 and finally by gel filtration according to Górska et al. (2016) [32]. Three fractions were obtained (I, II, III) (Figure S1) and analyzed by chemical analysis (sugar, methylation, and determination of absolute configuration analysis) and NMR spectroscopy. The sugar and absolute configuration determination analysis showed that all fractions are composed of d-glucose residues with pyranose ring (d-Glc*p*). Methylation analysis of fraction I revealed the presence of 4-substituted glucopyranose (1,4,5-tri-*O*-acetyl-2,3,6-tri-*O*-methyl-d-glucitol-1-*d*).

In fraction III, we identified the 6-substituted glucopyranose (1,5,6-tri-*O*-acetyl-2,3,4-tri-*O*-methyl-d-glucitol-1-*d*), 3,6-disubstituted glucopyranose (1,3,5,6-tetra-*O*-acetyl-2,4-di-*O*-methyl-d-glucitol-1-*d*), 3-substituted glucopyranose (1,3,5-tri-*O*-acetyl-2,4,6-tri-*O*-methyl-d-glucitol-1-*d*) and terminal glucopyranose (1,5-di-*O*-acetyl-2,3,4,6-tetra-*O*-methyl-d-glucitol-1-*d*) in the molar ratios 2:0.4:1.8:1.8. In fraction II, which is a mixture of both I and III fractions, we identified all of the above-mentioned monosaccharide derivatives.

A complete structural analysis was performed by two-dimensional (2D) NMR spectroscopy on all fractions (I-III). The ^1^H-^1^H COSY spectra allowed for the identification of H-2 signals of the residues and subsequently, the ^1^H-^1^H total correlation spectroscopy (TOCSY) spectra with different mixing times and the ^1^H-^13^C Heteronuclear Single Quantum Coherence spectroscopy (HSQC-DEPT) spectra allowed for the assignment of the H-3 to H-6, 6′ signals for each residue of three fractions. To avoid repetition, structural analysis of fraction II was described in detail in the text (it is a mixture of polysaccharides presented in both I and III fractions).

**Figure 1:**
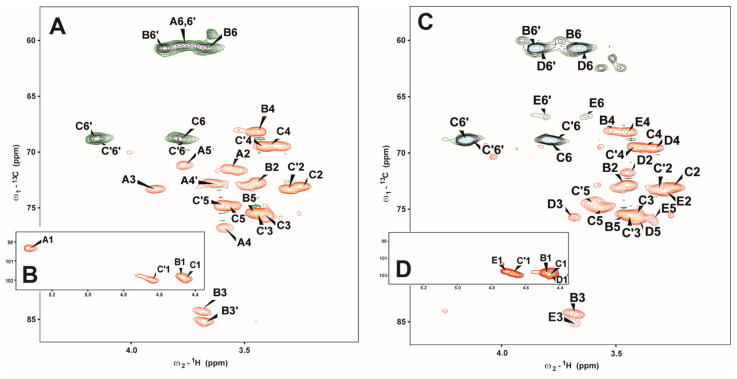
Selected parts of the ^1^H-^13^C HSQC-DEPT NMR spectra of fraction II (**A**,**B**) and fraction III (**C**,**D**).

The HSQC-DEPT spectrum of fraction II contained signals for four anomeric protons and carbons, respectively (Figure 1A,B and Table 1).

Residue **A** (δ_H_/δ_C_ 5.33/99.7 ppm, ^1^*J*_C-1,H-1_ ~172 Hz) was recognized as the 4-substituted α-d-Glc*p* based on the characteristic proton spin system. The downfield shift value of C-4 (δ_C_ 76.7 ppm) indicated that this is a 4-substituted residue [32,33].

Residues **B** (δ_H_/δ_C_ 4.48/102.6 ppm, ^1^*J*_C-1,H-1_ ~165 Hz) was recognized as the 3-substituted β-d-Glc*p* based on the basis of the large vicinal couplings between all protons in the sugar ring and the characteristic high chemical shifts of the C-3 at δ_C_ 84.5 ppm signals.

Residue **C** (δ_H_/δ_C_ 4.46/103.0 ppm, ^1^*J*_C-1,H-1_ ~163 Hz) and residue **C’** (δ_H_/δ_C_ 4.63/103.0 ppm, ^1^*J*_C-1,H-1_ ~163 Hz) were recognized as the 6-substituted β-d-Glc*p* based on the large vicinal couplings between all protons in the sugar ring and characteristic for substitution chemical shifts of C-6 signals at δ_H_/δ_C_ 3.80, 4.15/68.8, and at δ_H_/δ_C_ 3.77, 4.13/68.9 ppm, for **C** and **C’**, respectively. The significant difference in chemical shift values was observed only for anomeric protons of **C** and **C’** residues at δ_H_ 4.46 and 4.63 ppm, respectively. The presence of two→6-β-d-Glc*p* residues in fraction II can be explained by fraction heterogeneity, the presence of a mixture of three polysaccharide structures.

The monosaccharide sequences in fraction II were established using a ^1^H-^1^H nuclear Overhauser effect spectroscopy (NOESY) and ^1^H-^13^C Heteronuclear Multiple-Bond Correlation spectroscopy (HMBC) experiments. NOESY spectra showed strong inter-residue cross-peaks between the following transglycosidic protons: H-1 of **A**/H-4 of **A**, H1 of **B**/H3 of **B**, H-1 of **C**/H-6 of **C**, and H-1 of **C’**/H-3 of **B**. The HMBC experiment of fraction II confirmed substitution positions of all monosaccharide residues except for the glycosidic bond between **B** residues in β(1→3) glucan.

Each of →6-β-d-Glc*p* (**C** and **C’**) residues belong to different polysaccharide structures presented in fraction II. While the **C** residue builds the main β(1→6)-glucan, the **C’** residue substitutes **B** residue in position 3 in another polysaccharide–β(1→3) glucan, presented in fraction II. The amount of **C’** residue in comparison with **B** is significantly lower.

**Figure 2:**
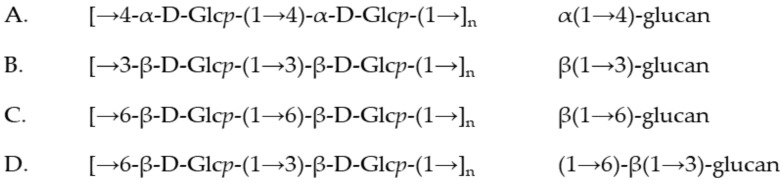
The structure of polysaccharides presented in fraction II (*A/B-C*) separated from *Se-Le-30* crude extract: (**A**) α(1→4) glucan; (**B**) β(1→3) glucan; (**C**) β(1→6) glucan; (**D**) β(1→6)-β(1→3) glucan.

The fractions obtained after separation of crude extract of *Se-Le-30* were successive: (I) fraction of homopolysaccharide α(1→4) glucan; (II) fraction a mixture of shorter chains of α(1→4) glucan, β(1→6) glucan, β(1→3) glucan and also β(1→6)-β(1→3)-glucan; (III) fraction contains a mixture of shorter chains of β(1→6) glucan, β(1→3) glucan and β(1→6)-β(1→3)-glucan, respectively (Figure 2). Simultaneously, signals of 3,6-disubstituted β-d-Glc*p* (residue **E**) and β-d-Glc*p* (residue **D**) have been identified (Figure 2C,D); however, the sequence of these branched molecules was not determined. It has to be pointed that in fraction III, the β(1→6) and β(1→3) glucans dominated. Residues **E** and **D** have been observed in fractions II, and III in methylation analysis.

### 2.3. Homogeneity and Molar Mass Determination of the Lentinan Analog (Se-Le-30) and Isolated Polysaccharides

**Figure 3:**
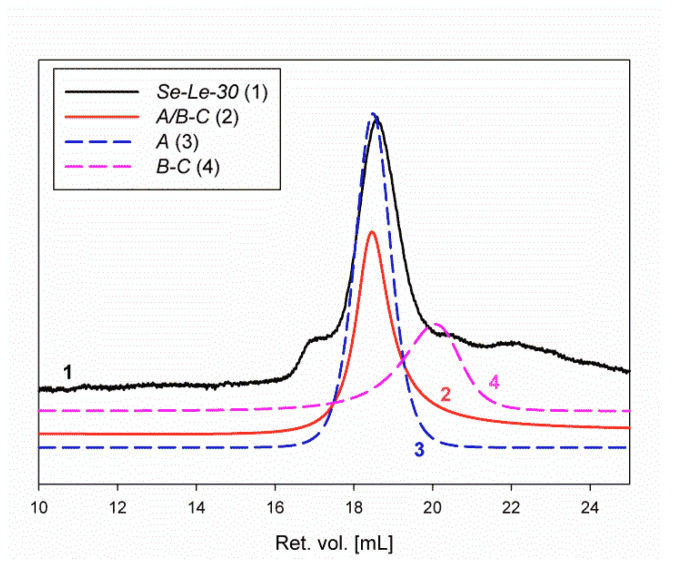
Refractive index (RI) traces from SEC of the *Se-Le-30*, *A* (I), *A/B-C* (II), and *B*-*C* (III) fractions. The lines for the *A*, *A/B-C*, and *B*-*C* fractions were smoothed to remove noise.

RI traces from SEC of fractions *Se-Le-30*, *A* (I), *A/B-C* (II), and *B*-*C* (III) are shown in Figure 3. The respective RALS traces of these products are shown in Figure S2.

Additional SEC traces from the RI, light scattering (LS), and viscosity detectors are provided in the Supporting Information (Figure S3). Bimodal signals were observed from the LS detectors for all analyzed samples. A bimodal signal for RI was observed only for the *Se-Le-30* fraction (Figure S3a). For all analyzed samples, both LS signals were shifted to the lower values of retention volume (higher molar masses) in relation to the RI and viscometer signals. The signals measured from the four detectors for each product do not fully overlap, resulting in a broader distribution of the molar masses (Table S1 in Supplementary Materials).

The molar masses for the analyzed products were determined by OmniSEC for the polysaccharide fractions as follows: *Se-Le-30*: M_n_ = 1.69 × 10^6^ g/mol, M_w_ = 3.62 × 10^6^ g/mol, *Ð* = 2.15 (dispersity); *A/B-C*: M_n_ = 1.72 × 10^6^ g/mol, M_w_ = 2.18 × 10^6^ g/mol, *Ð* = 1.27; *A*: M_n_ = 1.82 × 10^6^ g/mol, M_w_ = 2.25 × 10^6^ g/mol, *Ð* = 1.24; and *B*-*C*: M_n_ = 5.06 × 10^4^ g/mol, M_w_ = 1.10 × 10^5^ g/mol, *Ð* = 2.18. Fraction *B*-*C* had a much lower molar mass. Broad traces were observed for RI and viscosity, as well as bimodal traces for LS detectors. The character of the SEC curves suggests that the *B*-*C* fraction does not have a uniform structure. We assume that it is a mixture of linear and branched polysaccharides. The behavior of the *B*-*C* fraction is in agreement with its molecular modeling.

The *A*, *A/B-C*, and *B*-*C* fractions were isolated and purified on chromatographic columns. Unfortunately, this procedure did not ensure the isolation of completely pure products without any impurities. A more detailed analysis of the RI traces for the *Se-Le-30, A,* and *A/B-C* fractions (after the deconvolution procedure; Figure S3) allowed us to determine the content of the remaining fraction, *B*-*C*. Product *A* is the main component of the *Se-Le-30* fraction, and product *B*-*C* (with much lower molar mass) is present in only small quantities. Thus, in the *Se-Le-30* fraction, the *A* and *B*-*C* content were equal to 77.86% and 2.86%, respectively (Figure S3). In fraction *A/B-C,* a higher amount of *A* (85.35%) and *B*-*C* (4.23%) was observed (Figure S4). Finally, deconvolution of the RI signal of product *A* allowed for the determination of the residual content of the *B*-*C* fraction to be 2.73% (Figure S3). The overall purity of fraction *A* was estimated and equal to 86.48%. These calculations should be considered with caution because of the great difference in the content of both products.

**Figure 4:**
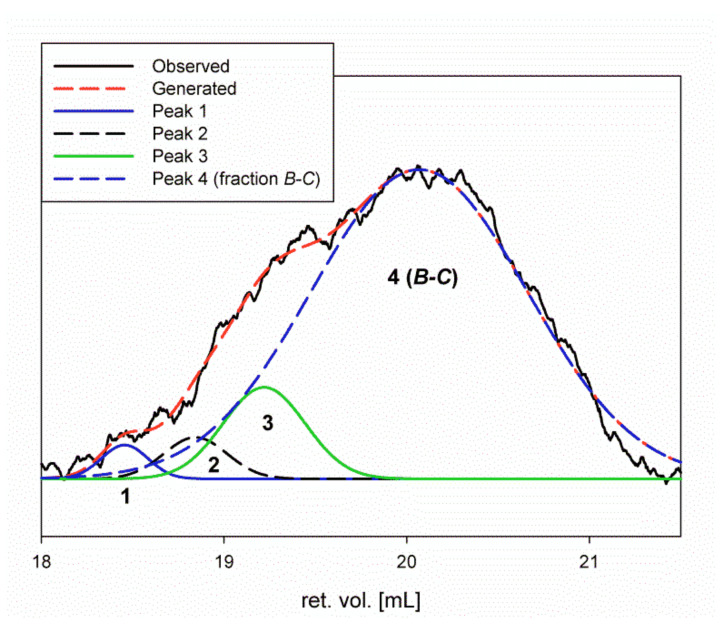
Deconvolution of the RI signal of product *B*-*C*.

Deconvolution of the RI signal of product *B*-*C* allowed the overall purity of fraction *B*-*C* to be determined to be 85.03%. We were also able to estimate the content of three other products (peaks 1–3) with slightly higher molar masses: 2.03% (18.5 mL), 3.32% (18.8 mL), and 9.63% (19.2 mL), respectively (Figure 4).

In addition, based on linear calibration to β-glucans, the following molar masses were calculated for the respective peaks: 96,500 g/mol (peak 1), 74,000 g/mol (peak 2), 56,700 g/mol (peak 3), 31,300 g/mol (fraction *B*-*C*).

### 2.4. Molecular Modeling

**Figure 5:**
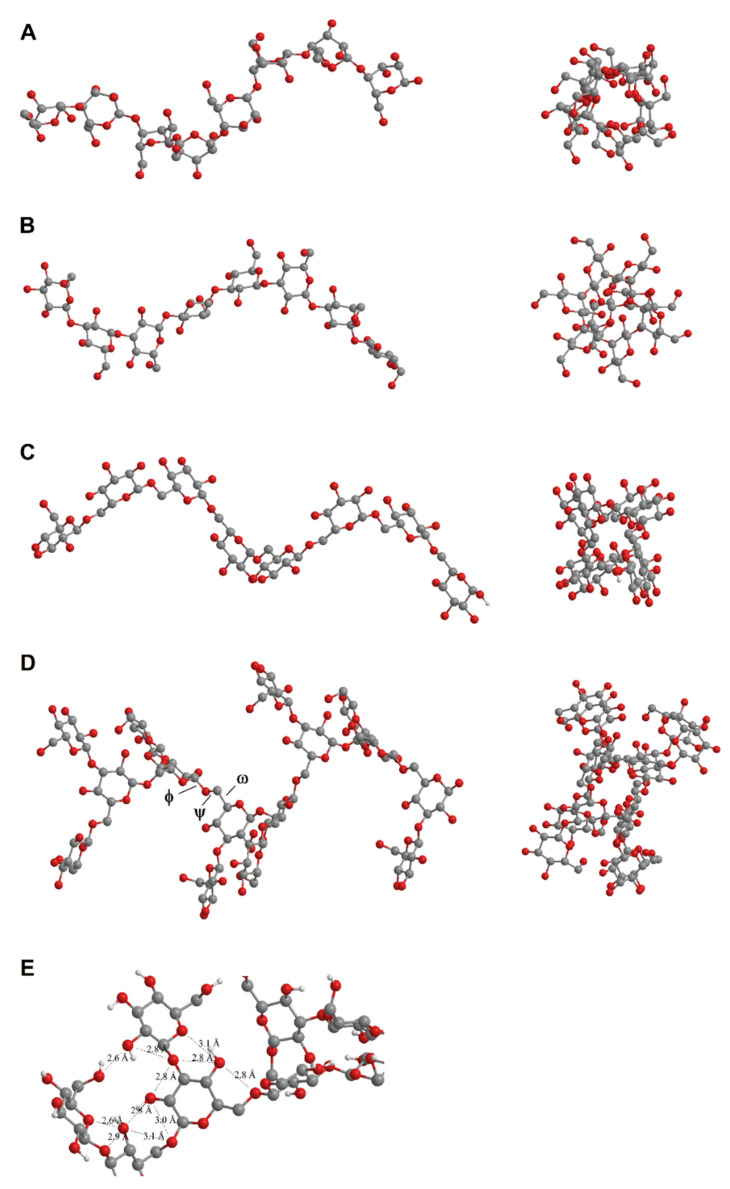
Structural models of the investigated polysaccharides; side view (left) and a view along the axis (right). (**A**) Polysaccharide *A* (1,4-α-d-glucan). (**B**) 1-3-β-d-glucan - component of *B*-*C* fraction. (**C**) 1-6-β-d-glucan-component of *B*-*C* fraction. (**D**) 1-3-β-branched 1-6-β-d-glucan - component of *B*-*C* fraction. (**E**) Distances (Å) between oxygen atoms, suggesting an opportunity for stabilization of the helical structure of 1-3-β-branched 1-6-β-d-glucan by hydrogen bonds. Created using the Chem3D Pro module of ChemOffice Professional 2017 (PerkinElmer). Energy minimization was performed using the MM2 method.

The MM2 calculations showed that all components of polysaccharide fractions, *A* and *B*-*C*, tend to adopt wide helical conformations (Figure 5). Polysaccharide *A* was a linear 1,4-α-d-glucan and was expected to adopt an amylose-like structure (Figure 5A). We previously described the deformation of the helical structure by the replacement of the oxygen atom in the glycosidic bond with Se [27]. However, Se incorporation cannot affect the overall structure of the polysaccharide *A* chain because of the very low selenium content in the samples tested; the Se incorporation effect is only local. Being an amylose-like polysaccharide, *A* was expected to form left-handed helices with six glucose units per turn and stabilized by hydrogen bonds.

According to the MM2 calculations, the α-helix was also the most probable conformation in the 1-3-β-d-glucan, 1-6-β-d-glucan and 1-3-β-branched 1-6-β-d-glucan, components of the polysaccharide fraction *B*-*C* (Figure 5B–D). However, in contrast to 1-3-β-ducan, the helical structure of 1-6-β-d-glucan and 1-3-β-branched 1-6-β-d-glucan may be flexible.

One reason is an additional degree of freedom arising from the link ω (C_6_-C_5_ in 1-6-glucans) compared to 1-3-, 1-4-, and 1-2-glucans, in which only φ (C_1_-O) and ψ (O-C_3_, O-C_4_, O-C_2_) linkages are present. The second reason is the limited possibility of creating intramolecular hydrogen bonds (Figure 5D).

To investigate whether the presence of 1-3-β branches affects the adopted conformation of polysaccharide *B*-*C*, we performed the MM2 experiments for its 1-3-β unbranched counterpart (Figure 5C,D). The results show similar conformations for both branched and unbranched polysaccharides. The models do not take into account the phenomenon of slight chain deformation due to the introduction of selenium atoms into the molecule described in our previous work, presenting only the general spatial arrangement of the molecule.

### 2.5. Effects of Polysaccharides on the Proliferation of Human Peripheral Blood Mononuclear Cells

**Figure 6:**
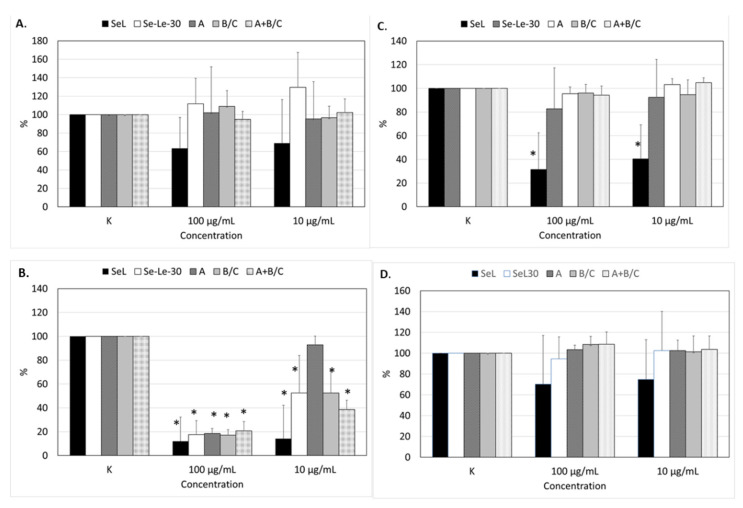
The effects of polysaccharides (*Se*-*L*, *Se-Le-30*, *A*, *B*-*C*, and *A/B-C)* on the proliferation of peripheral blood mononuclear cells (PBMCs). (**A**) Non-stimulated (autostimulation). (**B**) Stimulated with anti-CD3 monoclonal antibody (OKT3). (**C**) Stimulated with phytohemagglutinin (PHA). (**D**) Stimulated with the suspension of *Staphylococcus aureus* Cowan strain (SAC). The results are presented as the percentage of control (without polysaccharides) proliferation. * *p* < 0.05 versus control (K). The error bars correspond to the standard deviation. The results for the *Se*-*L* fraction were described by Kaleta et al. (2019) [30].

The effect of the *Se-Le-30* fraction (lentinan analog) and fractions *A* and *B*-*C* on non-stimulated, OKT3-, phytohemagglutinin (PHA-), and *Staphylococcus aureus* Cowan (SAC)-stimulated peripheral blood mononuclear cell (PBMC) proliferation is presented in Figure 6. The results are compared to those previously obtained for the *Se*-*L* fraction [27].

As reported previously [30] for the *Se*-*L* fraction, autostimulation showed some tendency to decrease the proliferation of PMBCs. The opposite tendency was noted for the *Se-Le-30* fraction, but these effects were not significant (Figure 6A). The *Se*-*L* fraction demonstrated some tendency to decrease the proliferation of PMBCs stimulated with SAC [30], but this effect was not observed for the *Se-Le-30* fraction isolated by the Chihara method [34] and the purified polysaccharides *A* and *B*-*C* (Figure 6D).

The OKT3-stimulated T-cell proliferation was significantly inhibited by all tested fractions (*Se-Le-30*, *A*, *B*-*C*, and *A/B-C*) at 100 µg/mL, but at a concentration of 10 µg/mL, this effect was shown only in the *Se-Le-30*, *B*-*C*, and *A/B-C* fractions. For the *Se-Le-30* fraction, the degree of inhibition at 100 and 10 μg/mL was approximately 82% and 48%, respectively. For polysaccharides *A* and *B*-*C*, the degree of inhibition at 100 µg/mL was approximately 82% and 83%, respectively. However, the difference between the fractions was significant at 10 µg/mL; fraction *A* was practically inactive, whereas the degree of inhibition in fraction *B*-*C* was 47%. The relatively high standard deviations in the *Se*-*L* and *Se-Le-30* fractions (Figure 6) were due to the diverse response of the lymphocyte cultures from different donors to the mitogen. No significant inhibitory effect of polysaccharides *A* and *B*-*C* and mixture *A/B-C* on PHA-stimulated PBMCs was observed (Figure 6C).

For fraction *A/B-C*, the impact on OKT3-stimulated PBMC proliferation was significantly stronger than for polysaccharides *A* and *B*-*C* separately (Figure 6). The cell viability assessment by trypan blue exclusion confirmed that the inhibitory effect on OKT3- and PHA-stimulated PBMC proliferation was not due to the toxicity of the examined polysaccharides.

## 3. Discussion

The main goal of the current study was to investigate whether Se-polysaccharides are responsible for the immunosuppressive activity of previously isolated Se-polysaccharide-protein fraction *Se*-*L* [27,30]. Lentinan, the Japanese immunostimulatory drug for which we intended to obtain a selenized analog, was isolated for the first time by G. Chihara from the fruiting bodies of *L. edodes* in a multi-step fractionation procedure using strong bases and organic acids [34]. We predicted that during the isolation of Se-polysaccharides from *L. edodes* mycelium using this method, a significant portion of selenium may be lost. The selenium-containing polysaccharides (selenoglycosides) may be unstable in a strongly basic and acidic environment. Therefore, in our previous research, we chose the Yap and Ng [35] method of isolating lentinan. The advantage of this method is the mild conditions of the isolation process, resulting in higher polysaccharide yield and less loss of selenium. The disadvantage, however, is lower homogeneity and reduced purity of the obtained fraction versus the Chihara method (87% versus 96%).

As we wanted to exclude the immunosuppressive effect of the previously examined *Se*-*L* fraction being caused by components other than polysaccharides (proteins or other undefined impurities), we decided to obtain a pure lentinan analog via Chihara’s isolation method. To compensate for the loss of selenium during isolation, the mycelium culture was cultivated in a medium containing a higher concentration of selenium (30 ppm). By raising the concentration of selenium in the culture medium to 30 ppm, we achieved a very significant increase in the concentration of Se in the *L. edodes* mycelium: 1753 μg/g versus 1040 μg/g for the culture enriched in 20 ppm. We also observed a significant increase in the concentration of selenium in the *Se*-*L* fraction isolated according to the Yap and Ng method [35] (305 μg/g versus 190 μg/g). However, the lentinan analog *Se-Le-30* isolated by Chihara’s method contained a much lower concentration of selenium (48 μg/g).

Selenium content in the chromatographically purified fractions described below as fractions I (*A*), II (*A/B-C*), and III (*B*-*C*) was slightly lower than the *Se-Le-30* fraction (17, 22 and 38 µg/g, versus 48 µg/g), indicating the highest selenium content in the β-d-glucan fraction. Thus, a change in the isolation method resulted in a significant loss of selenium but also had an expected positive effect, a significant increase in the purity of the fraction in terms of protein and sugars other than glucose content. As we demonstrated previously by X-ray absorption fine structure (XAFS) spectral analysis in the near edge region (XANES) [27], selenium in the examined Se-polysaccharide structure was present at the –II oxidation state and was organically bound. The simulation analysis in the EXAFS region suggested that selenium is most likely bound by a glycosidic link in a β-1,3 or α-1,4-glycosidic bond. In the current study, we did not repeat these tests due to the low concentration of selenium in the chromatographically purified fractions, which was below the detection level of the methods for determining the chemical environment of selenium (XAS and ^77^Se-NMR).

As stated above, the main question that arose when analyzing the results of our previous study [30] was the underlying reason for the immunosuppressive effect of the *Se*-*L* fraction, which is opposite to the Japanese drug lentinan, which is isolated by the same method from the fruiting bodies of the same species of mushroom. Structural studies of the *Se*-*L* fraction indicate it contains a mixture of 1,4-α 1,6-β- and 1,3-β-glucans and mannans [27], whereas lentinan is a (1,6;1,3)-β-glucan [25]. The proportion of 1,6- and 1,3-β-glycosidic linkages in the mycelial fraction was also different from lentinan [27].

### 3.1. Se-Le-30 Fraction

Similar to the *Se*-*L* fraction [27], data on the type of glycosidic bonds were recorded for the *Se-Le-30* fraction. The NMR and TRISEC studies showed that the *Se-Le-30* fraction was not homogenous. Product A was the main component of the *Se-Le-30* fraction, and product B-C with a much lower molar mass, was present only in small quantities. The approximate mass ratio of the *A* and *B*-*C* polysaccharides in the *Se-Le-30* fraction was 26:1. In addition, the *Se-Le-30* fraction contained approximately 6% mannose and 4% protein by weight (perhaps in the form typical for mushroom cell wall mannoproteins). The selenium content in the *Se-Le-30* fraction was nearly fourfold lower than in the previously isolated *Se*-*L* fraction [27].

### 3.2. Fraction A (I)

The sugar analysis and absolute configuration determination showed that both isolated polysaccharides (*A* and *B*-*C*) were composed of d-glucose. Methylation analysis indicated that polysaccharide *A* was a linear homopolymer built of →4)-α-d-Glc*p*-(1→ residues. The data obtained on the structure of fraction *A* indicated that this natural polysaccharide of fungal origin, most likely a component of the *L. edodes* cell wall, had a structure similar to amylose, a polysaccharide of plant origin. Its molar mass determined by TRISEC was significantly higher than the average masses typical for amylose. According to Joye (2019) [36], the molar mass range of amylose is quite broad and varies between 8 × 10^4^ and 10^6^ g/mol depending on the plant species, varieties, and maturity of the starch under study.

The >80% content of *A*, an amylose-like α-1,4-d-glucan in the *Se-Le-30* polysaccharide fraction isolated from *L. edodes* mycelium, was surprising. The amylose-like polysaccharide has not yet been described as a main hot water-soluble component of the cell wall of *L. edodes*. However, McCleary and Draga (2016) [37] determined the α- and β-glucans in commercial preparations of fungal origin, including *L. edodes* extracts; they contain up to 80% α-glucans, instead of the β-glucans declared by the manufacturer. The authors explained the presence of α-glucans as an artefact caused by the cultivation and isolation method. They stated that commercial mushroom mycelia are cultivated over a sterilized cereal grain base. In the latter process, the mycelium-infested grain is harvested and the polysaccharide fractions isolated. Based on the analytical data, they suggested that the starch from the grain is the major α-glucan present in the final product [37].

This was possible with mycelium grown on a solid medium (grain). In our research, however, the mycelium was grown in a liquid medium that did not contain starch or solid components. When harvested, it was thoroughly filtered and rinsed, meaning that contamination with the culture medium was not possible. Polysaccharide *A*, a water-soluble 1,4-α-d-glucan, was a component of *L. edodes* mycelium.

The presence of 1,4-α-d-glucans in the cell wall of a fungus belonging to *Basidiomycota* was also found by Samuelsen et al. (2019) [14]. In hot water and hot alkali extracts from the edible mushroom *Albatrellus ovinus*, they detected a 1-4-α-ducan.

The secondary structure of polysaccharide *A* predicted based on molecular modeling was an α-helix (Figure 5A), analogous to amylose, which also tends to adopt a natural helical structure [36]. Natural polysaccharides have polydisperse molar mass, but using fractionation techniques, such as size exclusion chromatography, a low polydispersity index can be obtained. This is the case with fraction *A* having a low polydispersity index (*Ð* = 1.2), which indicates its homogeneity. The deconvolution of the RI signal of product *A* allowed for the determination of the residual content of the *B*-*C* fraction equal to 2.73%. The purification by GPC, in general, did not ensure the isolation of completely pure polysaccharides. In particular, the *B*-*C* fraction was very difficult to separate from fraction *A*, despite the very significant difference in molar mass, probably due to intermolecular interactions. A similar problem occurs when separating the amylose from amylopectin using gel-permeation chromatography. Perhaps amylose is cross-linked to amylopectin and, thus, eluted with amylopectin [38], or is interspersed with amylopectin molecules. Similar interactions may occur between polysaccharides *A* and *B*-*C*.

### 3.3. Fraction B-C (III)

The same analytical methods used to study the structure of fraction *A* (sugar, metylation and absolute configuration determination, analysis, and 2D NMR data) showed that the *B*-*C* fraction was a mixture of three glucans: 1,3-β-d-glucan, 1,6-β-d-glucan and β-1,3-branched 1,6-β-d-glucan. The molar mass determined by TRISEC was significantly lower than that of fraction *A*. The homogeneity of fraction *B*-*C* was also lower, which indicates a higher polydispersity index (*Ð* = 2.18).

1,6- β-Glucan has been shown to be an important component of *S. cerevisiae* and *C. albicans* cell walls [39,40,41]. However, last year, a number of publications reported the presence of 1-6-β-glucans in the cell wall of higher fungi [14,27,42]. The structural analysis of water and alkali extracts from *A. ovinus* fruit bodies suggested the presence of two different β-d-glucan backbone structures: a 1-6-linked β-d-glucan with single β-d-Glc*p* residues at *O*-3, and a (1→3)-linked β-d-glucan with branches [14]. The *B*-*C* polysaccharide of a similar structure was present in low proportions in the tested *Se-Le-30* fraction isolated from *L. edodes*, which was probably due to its low solubility in hot water. A similar compound was much more efficiently isolated by Samuelsen et al. (2019) [14] and by Li et al. (2019) [42] with hot alkali solution.

To summarize, lentinan, the polysaccharide isolated from the fruit bodies of *L. edodes* by G. Chihara was a β-d-glucan with 1-3-β-linked backbone and 1-6-β -linked side chains. The polysaccharide fraction isolated by the same procedure from the submerged cultured *L. edodes* mycelium proved to be a mixture of linear 1-4-α-d-glucans, 1-3- β-d-glucans, 1-6-β-d-glucans and branched 1-6-β-d-glucan with 1-3-β -linked backbones.

None of the analytical methods (chromatographic, methylation, and NMR) allowed to reliably detect the presence of selenium-containing monosaccharides, mainly due to their instability under the polysaccharide hydrolysis conditions and due to their low concentration in the analyzed fraction.

### 3.4. Biological Activity

In our previous study, we examined the effects of the isolated Se-polysaccharide-protein fraction (*Se*-*L*) on the proliferation of human PBMCs [30]. We used lymphocyte proliferation induced by OKT3 and PHA as a method to evaluate the T-cell proliferative response. The two mitogens use different mechanisms to promote T-cell effector functions; PHA stimulates T-cell proliferation via interactions with the *N*-acetylgalactosamine glycoprotein present on these cells [43], whereas OKT3 stimulates T cells via CD3-mediated signaling [44]. We also used lymphocyte proliferation induced by SAC to evaluate B-cell responsiveness. Here, we conducted the same experiments for the *Se-Le-30*, *A* (I), *B*-*C* (III)*,* and *A/B-C* (II) fractions. The results clearly demonstrate that, for the immunosuppressive activity of the previously described *Se*-*L* fraction [30], polysaccharides *A* and *B*-*C* were primarily responsible. The selenium content also had a significant impact on the activity.

The suppression of T-lymphocyte proliferation by currently isolated fractions was weaker but more selective than with the *Se*-*L* fraction (Figure 6). In higher concentrations (100 mg/mL), there was no significant difference between the activity of the *Se-Le-30, A, B-C,* and *A/B-C* fractions. At lower concentrations (10 µg/mL), however, the differences become significant. Polysaccharide *B*-*C* was almost twice as active as polysaccharide *A*. Interestingly, the mixture of polysaccharides *A* and *B*-*C* was more active than the individual fractions, suggesting possible synergism or the formation of active intermolecular structures.

The effect of selenium on the polysaccharide activity was clearly visible, particularly at lower concentrations (10 µg/mL). A fourfold decrease in the concentration of selenium in the *Se-Le-30* fraction versus *Se*-*L* correlated with an approximately fourfold decrease in the inhibition of T-cell proliferation.

Selective immunosuppressive activity is not typical for polysaccharides of fungal origin. The probable mechanisms for this are not known and have not been described. For the previously isolated *Se*-*L* fraction [27], we observed significant inhibition of the proliferation of the T lymphocytes, most likely via CD3 receptor, but the suppression was also seen in PHA-stimulated PBMCs, though the effect was significantly weaker [30]. In the autostimulation and SAC-stimulation settings, the *Se*-*L* fraction exhibited some tendency to decrease the proliferation of PMBCs, though it was not significant.

The suppression of T-cell proliferation for currently isolated fractions (*Se-Le-30, A, B-C*) in the OKT3 test, without an effect in the PHA test, suggests a mechanism via the CD3 receptors without interactions with the *N*-acetylgalactosamine glycoprotein. Therefore, we concluded that impurities in the *Se*-*L* fraction that are absent from currently examined polysaccharides were responsible for the interaction with B lymphocytes and the *N*-acetylgalactosamine glycoprotein.

We hypothesized that the completely different biological effect from lentinan depends, at least in part, on the selenium incorporation into the polysaccharide molecule, but also on the dissimilar structure of the fractions isolated from mycelial cultures versus lentinan [30]. Our current research confirmed this hypothesis. Lentinan is 1-6-β-branched 1-3-β-d-glucan with a M_w_ of 500 kDa. Its helical structure is stabilized by strong internal hydrogen bonding, reducing polysaccharide hydration. The structure is inflexible and rigid [25,45]. In contrast, the main component of the Se-enriched lentinan analogamylose-like polysaccharide *A* was a linear 1-4-α-d-glucan. It also has a helical structure but is much more flexible and hydrophilic than lentinan (1,3-β-d-glucan) [46]. This explains the good solubility and extractability of compound *A* (the polysaccharide with a M_w_ > 2000 kDa) in hot water.

Polysaccharide fraction *B*-*C*, the more active component of the *Se-Le-30* fraction (lentinan analog), was a mixture of unbranched 1-6-β-d-glucan, unbranched 1-3-β-d-glucan, and 1-3-β-branched 1-6-β-d-glucan – all with probable helical conformation (Figure 5). We found that numerous branches formed by single β-d-glucose molecules in 1-3-β-branched 1-6-β-d-glucan do not affect the adopted conformation (Figure 5C). However, the helical structure of two components of polysaccharide fraction *B*-*C* (unbranched 1-6-β-d-glucan and 1-3-β-branched 1-6-β-d-glucan) should be flexible and labile because of an extra degree of freedom in the link ω (C_6_-C_5_ in 1-6-glucans; Figure 5D) compared to 1-3-, 1-4-, 1-2- glucans. The distances between some oxygen atoms (2.8–2.9 Å) suggest an opportunity for stabilization of the helical structure by hydrogen bonds (Figure 5D). Low polysaccharide *B*-*C* content in the *Se-Le-30* fraction suggests low content in the *L. edodes* cell wall and/or low extractability with hot water. The latter is possible, as two papers have described the isolation of analogous 1-3-β-branched 1-6-β-d-glucan from mushroom cell walls using hot basic solution [14,42].

Elements of the polysaccharide structure determine their biological activity, and the activity-structure relationship is well known for 1,3-β-d-glucans. For other groups of fungal polysaccharides, this knowledge is poor [47]. Further investigation of the mechanism of action and the structure-activity relationships of Se-polysaccharides is challenging due to the unique biological activity and the unique structure as components of the cell wall of *L. edodes* mycelium. However, further studies are necessary due to their hypothetical use in therapy.

Notably, the results of our research do not indicate a lack of 1-6;1-3-β-d-glucans in the cell wall of Se-enriched mycelium from *L. edodes*. These compounds are likely characterized by worse hot water solubility than 1-3-β-d glucans isolated by Chihara (1970) [34].

The mushroom cell wall is a dynamic structure that is continuously evolving in response to environmental conditions [4]. The culture method, conditions, and medium composition may affect the structure of cell wall polysaccharides. The results of our current research show that even a well-known and widely described species of fungus in submerged culture in a specific liquid medium may biosynthesize a polysaccharide with a completely different structure and biological activity than described thus far.

## 4. Materials and Methods

### 4.1. Biosynthesis and Isolation of Se-Enriched Polysaccharide Fractions

#### 4.1.1. Mushroom Strain and Cultivation Conditions

The *Lentinula edodes* (Berk.) Pegler strain used in our study was American Type Culture Collection (ATCC) 48085 (ATCC, Manassas, VA, USA). The seed culture was grown under the conditions described in our previous reports [28,29,48]. Mycelia used as the raw material for extraction of polysaccharides were cultivated under submerged conditions in a 10-L fermenter (BioTec FL 110, Stockholm, Sweden). The culture media was fortified with 30 μg/mL selenium by the addition of sodium selenite (Na_2_SeO_3_, Sigma, Cell Culture Tested, Sigma, Saint Louis, MO, USA). The initial pH of the medium was 6.5. The medium was inoculated with 5% (*v*/*v*) seed culture and cultivated at 26 °C. Fermentation was performed for 10 days under the following conditions: aeration rate, 0.5 volume per minute (vvm); agitation speed, 200 rev/min; and working volume, 8 L. Mycelia were harvested by filtration, washed three times with distilled water, and freeze-dried.

#### 4.1.2. Extraction of the Se-Enriched Polysaccharide Fraction Using the Chihara Method

**Figure 7:**
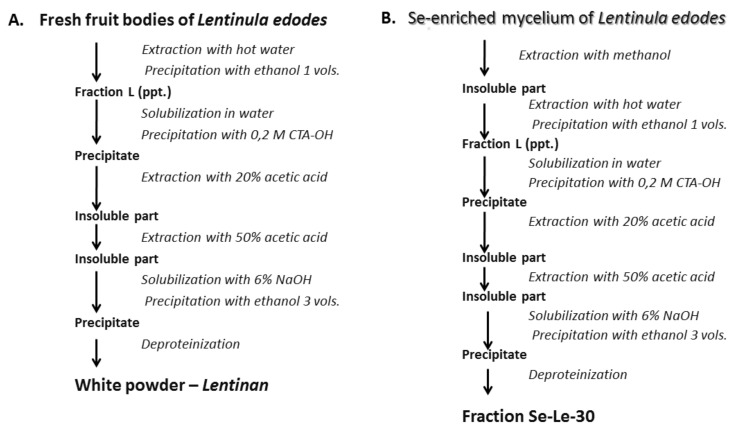
Isolation methods. (**A**) Chihara’s method of isolating lentinan. (**B**) Method modified for isolation of Se-enriched lentinan analogue (*Se-Le-30* fraction).

The Se-enriched polysaccharide fraction, the analog of the Japanese anticancer drug lentinan, was isolated from the Se-enriched *L. edodes* mycelium using the modified Chihara method [34]. The original method was completed by the preliminary extraction of lipids, small carbohydrate molecules, and other non-polysaccharide compounds in boiling methanol (1:4 *w*/*v*) for 4 h (Figure 7).

#### 4.1.3. Separation of Se-Enriched Polysaccharides Fraction Se-Le-30

The polysaccharides were purified according to Górska et al. (2016) [32]. Briefly, the freeze-dried *Se-Le-30* crude extract was purified by ion-exchange chromatography on a DEAE-Sephadex A-25 column (1.6 × 20 cm; Pharmacia Biotech, Piscataway, NJ, USA) and further by gel permeation chromatography on a Toyopearl HW-55S column (1.6 × 100 cm; Tosoh Bioscience LLC, Griesheim, Germany) fitted to an FPLC system (Amersham Pharmacia Biotech, Uppsala, Sweden). The fractions were eluted with a NaCl gradient (0–2 M in 20 mM Tris buffer, pH 8.2) and 0.1 M ammonium acetate buffer, respectively. The column effluents were monitored at 220, 260 and 280 nm with a UV-VIS absorbance detector and Knauer differential refractometer and for the carbohydrate content [49]. The fractions containing carbohydrates were collected.

### 4.2. Structural Analysis

#### 4.2.1. Molar Mass Determination by SEC Chromatography with Triple Detection

The molar mass of the analyzed fractions was estimated by size exclusion chromatography (SEC) with triple detection (TRISEC) as described in our previous report [27]. Namely, three TSK-GEL columns (G5000 PW_XL_ + 3000 PW_XL_ + 2500 PW_XL_; 7.8 × 300 mm; Tosoh) with a Knauer K-501 pump, a refractive index (RI) detector (LDC), and a double TDA 270 detector (laser light scattering [RALS + LALS] and viscosity detector; Viscotek, Houston, TX, USA) were used. The analysis was performed at 26 °C with a mobile phase of 0.1% NaN_3_ and a flow rate of 1.0 mL/min. The injection volume was 100 μL. Before injection, samples were filtered through 0.2-μm pore size membrane filters. OmniSEC software (Viscotek, USA) was used to calculate the number average molar mass (M_n_) for *dn*/*dc* = 0.145 (β-glucans in NaN_3_ solution). The procedure for calculating molar mass using OmniSEC software was reported previously [50].

#### 4.2.2. RP-HPLC Determination of Monosaccharide Composition

The monosaccharide composition of the polysaccharides was determined by a reversed-phase high-performance liquid chromatography (RP HPLC) method described previously [20]. Prior to the analysis, the polysaccharides were hydrolyzed for 5 h in 3M trifluoroacetic acid (TFA) at 120 °C.

#### 4.2.3. RP HPLC Determination of Se Content

The RP HPLC procedure to determine Se content was a modified fluorimetric method for Se determination after derivatization with 2,3-diaminonaphtalene (3,5-benzopiazselenol formation) with fluorescence detection as described previously [27,51].

#### 4.2.4. Determination of Protein Content in the Lentinan Analogue (*Se-Le-30*)

The protein content was determined using two independent methods as described previously [27]. The results of the well-known spectrophotometric Bradford method [52] were verified by measuring the nitrogen content in the analyzed fractions using a CHNS elemental analyzer (Vario EL III, Elementar, Germany). The protein content was calculated using a conversion factor of 6.25 for total nitrogen to protein.

#### 4.2.5. Sugar and Methylation Analysis and Determination of the Absolute Configuration

Polysaccharide samples (0.5 mg) were hydrolyzed with 10 M HCl at 80 °C for 25 min and evaporated under a stream of N_2_. The resulting monosaccharides were converted into alditol acetates, according to Sawardeker, Sloneker, and Jeanes (1956) [53]. For methylation analysis, the polysaccharide samples were permethylated according to the method described by Ciukanu and Kerek (1984) [54]. The product was purified by water-chloroform extraction. The methylated polysaccharides were hydrolyzed in 2 M TFA at 120 °C for 2 h and evaporated under N_2_. Finally, methylated monosaccharides were reduced with NaBD_4_ and acetylated for gas-liquid chromatography-mass spectrometry (GLC-MS) analysis using the same conditions as for the sugar analysis and butyl glycosides as described below. The absolute configurations of the sugars were determined by GLC-MS of their acetylated glycosides using (S)-/+2-butanol essentially as described by Gerwig, Kamerling, and Vliegenthart (1979) [55]. Alditol acetates and acetylated 2-butyl glycosides were analyzed by GLC-MS using an ITQ 700 Thermo Scientific system equipped with a ZB-5HT (Phenomenex) capillary column with a temperature gradient from 150 to 270 °C at 8 °C/min.

#### 4.2.6. NMR Analysis

NMR spectra were obtained on a Bruker 600 MHz Avance III spectrometer (Bruker, Billerica, MA, USA) using a 5-mm QCI ^1^H/^13^C/^15^N/^31^P probe equipped with a *z*-gradient. The NMR spectra were obtained for a ^2^H_2_O solution of the polysaccharide at 25 °C using acetone (δ_H_ 2.225, δ_C_ 31.05 ppm) as an internal reference. The polysaccharide (10 mg of fraction I and II and 2 mg of fraction III) was repeatedly exchanged with ^2^H_2_O with intermediate lyophilization. The data were acquired and processed using Bruker Topspin software (version 3.1) and SPARKY [56]. The signals were assigned using one-dimensional (^1^H, ^13^C, ^31^P) and two-dimensional (2D) experiments: correlation spectroscopy (COSY), total correlation spectroscopy (TOCSY), nuclear Overhauser effect spectroscopy (NOESY), ^1^H-detected heteronuclear single quantum coherence spectroscopy (HSQC) with and without carbon decoupling, HSQC-TOCSY, and ^1^H-^13^C heteronuclear multiple-bond correlation spectroscopy (HMBC). The TOCSY experiments were carried out with mixing times of 30, 60, and 100 ms, NOESY with mixing times of 100 ms and 300 ms, and HMBC with a mixing time of 60 ms.

### 4.3. Molecular Modeling

Structural models of the investigated polysaccharides were built using the Chem3D Pro module of ChemOffice Professional 2017 (PerkinElmer, Waltham, MA, USA). The energy minimization was performed using the MM2 method implemented in the software.

### 4.4. Effects of Polysaccharides on Human Peripheral Blood Mononuclear Cell Proliferation

To investigate the effect of polysaccharide fractions and isolated polysaccharides on human lymphocyte proliferation, we used a previously described method [30]. Briefly, peripheral blood mononuclear cells (PBMCs) were isolated from the heparinized blood of healthy donors by density-gradient centrifugation on a Histopaque-1077 (Sigma, Saint Louis, MO, USA). PBMCs were cultured in Parker medium (Biomed, Lublin, Poland) supplemented with 2 mM L-glutamine (Sigma, Saint Louis, MO, USA), 0.1 mg/mL gentamycin (KRKA, Novo Mesto, Slovenia), β-mercaptoethanol (Sigma, Saint Louis, MO, USA), 0.23% Hepes (Sigma, Saint Louis, MO, USA), and 10% heat-inactivated fetal bovine serum (FBS, Gibco, Thermo Fisher Scientific, Waltham, MA, USA).

The polysaccharide fractions from *L. edodes* (*Se*-*L*, *Se-Le-30*, I (*A)*, II (*A*/B-C) and III (*B*-*C*) were diluted in 0.9% NaCl (Fresenius Kabi, Bad Homburg, Germany) to achieve concentrations of 0.1–0.001 mg/mL. PBMCs were seeded into flat-bottom 96-well microplates at a density of 1 × 10^6^ cells/well and stimulated with specific T- and B-cell mitogens: anti-CD3 monoclonal antibody (mAb) (OKT3, 1 µg/mL, T-cell mitogen, BD Pharmingen, San Diego, CA, USA), phytohemagglutinin (PHA, 20 µg/mL, T-cell mitogen, Sigma, Saint Louis, MO, USA), and a suspension of *Staphylococcus aureus* Cowan strain (SAC, 0.004% *w*/*v*, B-cell mitogen, Calbiochem, San Diego, CA, USA). Polysaccharides were added to cell cultures in 100 µL aliquots of the prepared dilution per well. Control cultures contained an equivalent amount of 0.9% NaCl. As an internal control, an analogous test was carried out without any mitogens (autostimulation). PBMCs were cultured for 72 h at 37 °C in a humidified atmosphere with 5% CO_2_, pulsed with 1 *μ*Ci/well of [^3^H]-thymidine (113 Ci/nmol, NEN) for 18 h, and harvested using a Skatron cell harvester. Thymidine incorporation into the DNA of proliferating cells was analyzed in a liquid scintillation counter (Wallac Microbeta, Perkin Elmer, Waltham, MA, USA). Results were expressed as counts per minute (cpm). All experiments were performed in triplicate.

### 4.5. Statistical Analysis

The Mann–Whitney U-test and Spearman correlation were applied using Statistica 9.0 (StatSoft Inc., Tulusa, Ok, USA). Differences from control cultures were considered significant at *p* < 0.05.

## 5. Conclusions

The results of this study show that no uniformity or predictability is found in the structural features or functional characteristics of bioactive polysaccharides; the biosynthetic pathways, the structure of the polysaccharides obtained using the same mushroom species, and the bioactivity mechanisms are highly variable and confusing to researchers.

Currently, the substantial challenge and topic for further research are to develop the mechanism of selective suppression of T-cell proliferation by the isolated Se-polysaccharides.

The research results obtained thus far are currently being prepared for separate publication due to their size. The other important issue is the development of an isolation method for Se-polysaccharide that will not result in selenium loss, as well as the search for the optimal proportion of polysaccharides *A* and *B*-*C* in the active mixture.

## 6. Patents

The result of the described research is a patent application P.438570 “Selenized bioactive polysaccharide fraction from *Lentinula edodes*, pharmaceutical composition comprising selenized bioactive polysaccharide fraction, the selenized bioactive polysaccharide fraction for use as medicaments and a method of preparation thereof” filed with the Patent Office of the Republic of Poland on 22 July 2021.

## Figures and Tables

**Table 1:** ^1^H and ^13^C NMR chemical shifts of fraction II separated from *Se-Le-30* crude extract.

Sugar Residue	Chemical Shifts (ppm)					
	H1/C1	H2/C2	H3/C3	H4/C4	H5/C5	H6, H6′ C6
**A** →4)-α-d-Glc*p*-(1→	5.33 99.7	3.54 71.6	3.90 73.4	3.57 76.7	3.77 71.2	3.77, 3.80 60.5
**B** →3)-β-d-Glc*p*-(1→	4.48 102.6	3.45 72.8	3.69 84.5	3.48 69.2	3.46 75.5	3.67, 3.85 60.7
**C** →6)-β-d-Glc*p*-(1→	4.46 103.0	3.26 73.1	3.39 75.8	3.40 69.4	3.56 74.8	3.80, 4.15 68.8
**C’** →6)-β-d-Glc*p*-(1→	4.63 103.0	3.31 73.3	3.44 75.4	3.40 69.5	3.56 74.8	3.77, 4.13 68.9

Spectra were obtained for ^2^H_2_O solutions at 25 °C, and acetone (δ_H_ 2.225, δ_C_ 31.05 ppm) was used as an internal reference.

## Data Availability

The data presented in this study are available on request from the corresponding author and co-authors.

## Supplementary Materials

The following are available online. Table S1: SEC analysis (RI,RALS, LALS, and visc) of analyzed products; Figure S1: RALS traces for *Se-Le-30* and fractions *A*, *A/B-C*, and *B*-*C*; Figure S2: SEC traces. SEC traces. (a) *Se-Le-30*; (b) *A/B-C* fraction; (c) *A* fraction; (d) *B*-*C* fraction; Figure S3. Deconvolution of the Se-Le-30 RI signal; Figure S4. Deconvolution of the RI signal of product *A/B-C*.

## Sample Availability

Sample of the selenopolysaccharides are available from the authors.
