# Supplementary material for: Identification of the Primary Structure of Selenium-Containing Polysaccharides Selectively Inhibiting T-Cell Proliferation
Source: Molecules. 2021 Sep 6;26(17):5404. doi: 10.3390/molecules26175404 (PMC8434567; doi:10.3390/molecules26175404)
Supplement: Supplementary file 1 [file molecules-26-05404-s001.zip › molecules-1350326-supplementary.pdf]

Supporting Information

Table S1.

SEC analysis (RI, RALS, LALS, and visc) of analyzed products

Sample	Ret. vol. [mL]	M_n	M_w	$Đ$	Remarks
Se-Le-30	18.58	1.69×10^6	3.62×10^6	2.15	Bimodal signal for all detectors
A/B-C	18.48	1.72×10^6	2.18×10^6	1.27	Bimodal signal for LS and visc detectors
A	18.49	1.82×10^6	2.25×10^6	1.24	Bimodal signal for LS detectors
B-C	20.05	5.06×10^4	1.10×10^5	2.18	Bimodal signal for LS detectors

The OmniSEC calculation was based on glucan standard (glu-245). $Đ$ (dispersity) represents M_w/M_n

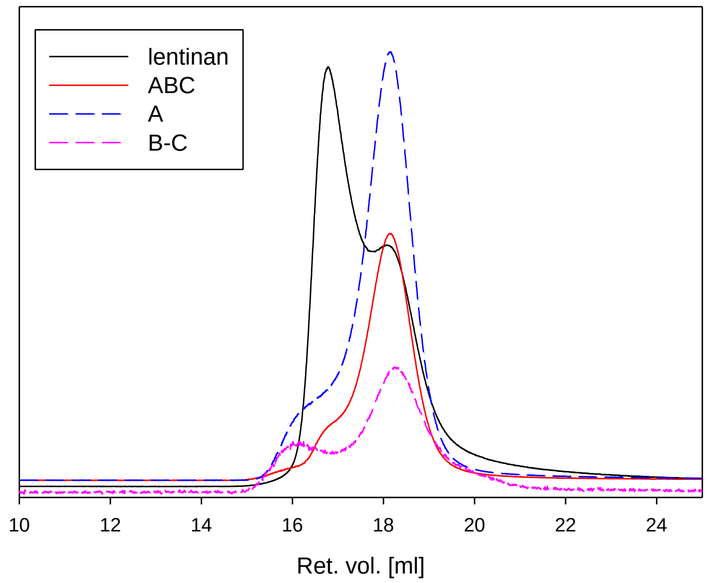

Figure S1. RALS traces for *Se-Le-30* and fractions *A*, *A/B-C*, and *B-C*.

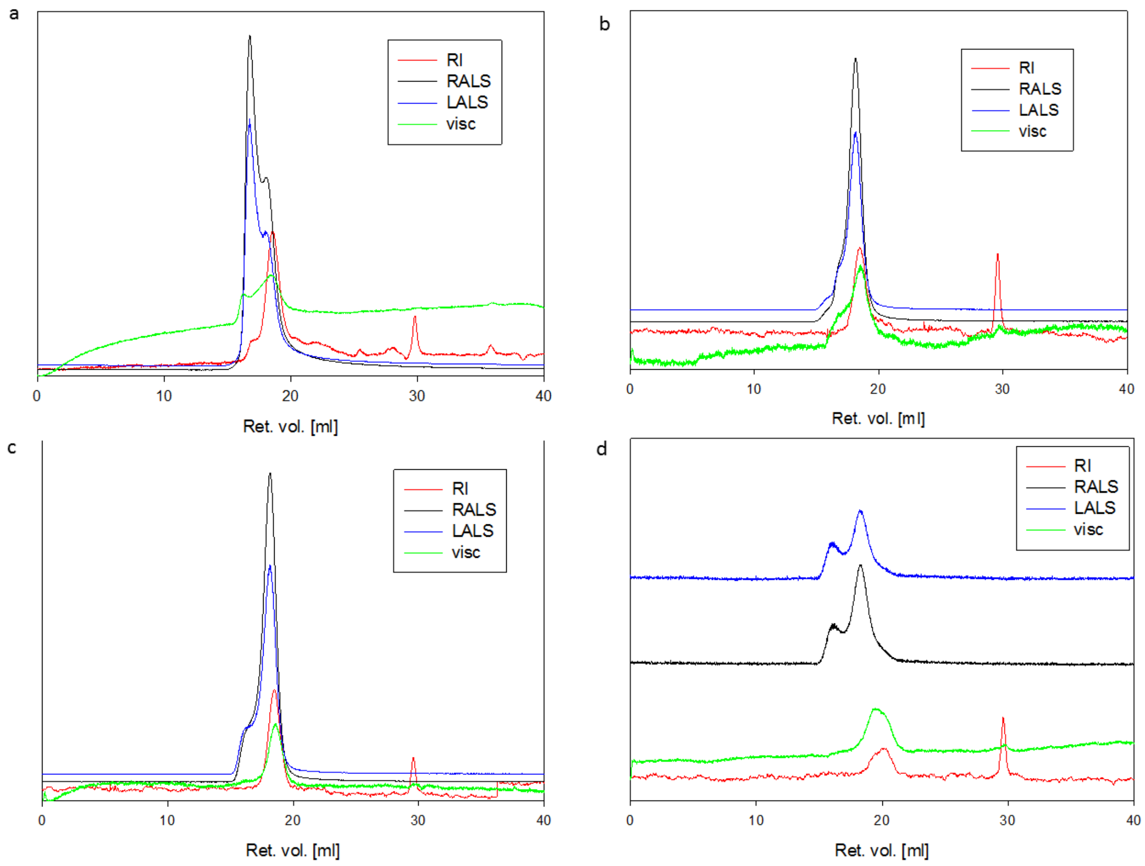

Figure S2. SEC traces. (a) *Se-Le-30*; (b) *A/B-C* fraction; (c) *A* fraction; (d) *B-C* fraction

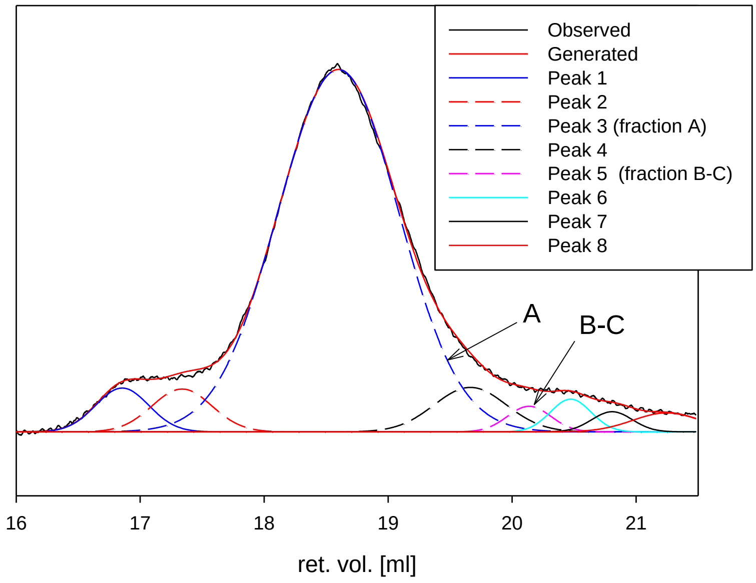

Figure S3. Deconvolution of the *Se-Le-30* RI signal. Curves corresponding to fractions *A* and *B-C* are marked by arrows.

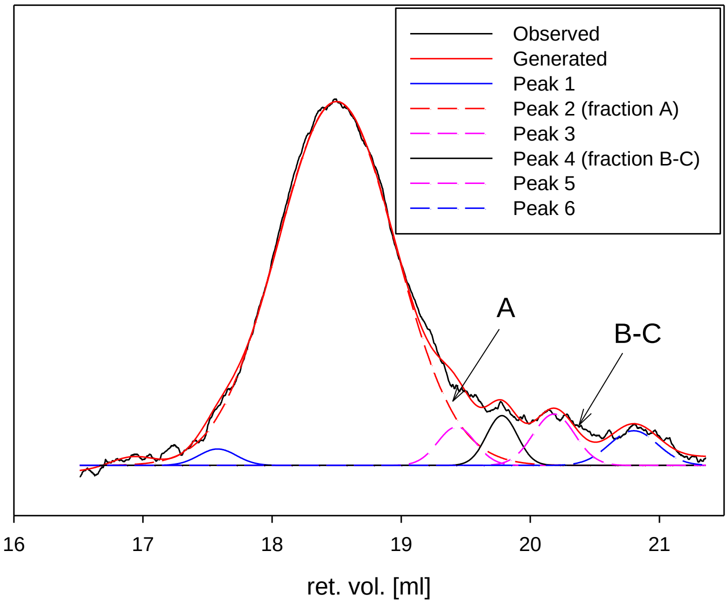

Figure S4. Deconvolution of the RI signal of product *A/B-C*. Curves corresponding to fractions *A* and *B-C* are marked by arrows.

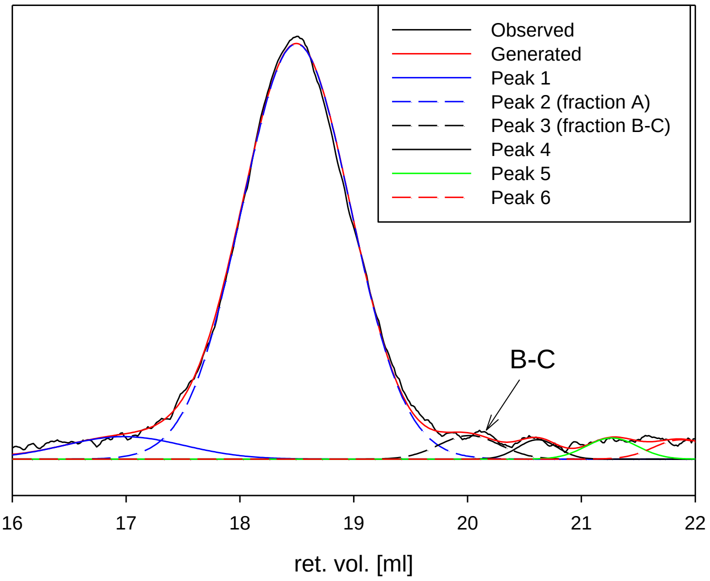

Figure S5. Deconvolution of the RI signal of product *A*. The curve corresponding to fraction *B-C* is marked by an arrow.
